# Severe congenital neutropenia due to jagunal homolog 1 (*JAGN1*) mutation: a case report and literature review

**DOI:** 10.3389/fped.2023.1223191

**Published:** 2023-07-17

**Authors:** Sanya Thomas, Geoffrey Guenther, Jared H. Rowe, Craig D. Platt, Akiko Shimamura, Ofer Levy, Lakshmi Ganapathi

**Affiliations:** ^1^ Precision Vaccines Program, Department of Pediatrics, Boston Children’s Hospital, Boston, MA, United States; ^2^ Harvard Medical School, Boston, MA, United States; ^3^ Division of Infectious Diseases, Boston Children’s Hospital, Boston, MA, United States; ^4^ Division of Hematology, Boston Children’s Hospital and Division of Pediatric Oncology, Dana-Farber Cancer Institute, Boston, MA, United States; ^5^ Division of Immunology, Boston Children’s Hospital, Boston, MA, United States; ^6^ Division of Hematology/Oncology, Boston Children's Hospital and Department of Pediatric Oncology, Dana-Farber Cancer Institute, Boston, MA, United States; ^7^ Broad Institute of MIT & Harvard, Cambridge, MA, United States; ^8^ Division of Pediatric Global Health, Massachusetts General Hospital, Boston, MA, United States; ^9^ Division of Pediatric Infectious Diseases, Massachusetts General Hospital, Boston, MA, United States

**Keywords:** *JAGN1*, congenital, neutropenia, children, severe congenital neutropenia (SCN)

## Abstract

Severe congenital neutropenia caused by jagunal homolog 1 (*JAGN1*) mutation is a rare condition resulting from maturation arrest secondary to endoplasmic reticulum stress response from impaired neutrophil protein glycosylation. Here, we report a case of a 4-year-old boy who presented with a history of recurrent infections and manifestations, including recurrent intracranial hemorrhage. A review of similar cases reported in the literature indicates that a bleeding diathesis has not been previously described in these patients. We hypothesize that this newly described association of bleeding complications in this patient with *JAGN1* mutation is secondary to defective glycosylation in the normal functioning of platelets or clotting factors. Recurrent infections with intracranial hemorrhage, new focal neurologic defects, or altered mental status in a child should warrant a suspicion for this immunodeficiency for the prompt initiation of treatment and prophylaxis for life-threatening infections or trauma.

## Background

Neutrophils are key effectors of innate immunity that contribute to defense against bacterial and fungal infections (1). Neutropenia is usually acquired, resulting from increased destruction, granulocyte apoptosis, or decreased granulocyte production. Severe congenital neutropenia (SCN) is a rare primary immunodeficiency resulting in recurrent infections (2). Patients with congenital neutropenia are at an increased risk of acquiring myelodysplastic syndrome or acute myeloblastic leukemia (3). Jagunal homolog 1 (JAGN1) is a protein important for neutrophil maturation and survival (4). Mutations in the gene encoding JAGN1 are a rare and recently identified cause of SCN characterized by recurrent infections along with bony abnormalities and a heterogeneous clinical presentation (3).

Here, we report a case of a patient with autosomal recessive JAGN1 deficiency leading to SCN, with recurrent infections associated with bleeding manifestations. To our knowledge, bleeding manifestations associated with JAGN1 deficiency have not been previously reported, and this may be the first case of JAGN1 neutropenia related to biallelic pathogenic heterozygous mutations. We also review the literature for other cases of congenital neutropenia resulting from JAGN1 deficiency.

## Case presentation

A 4-year-old boy presented with high-grade fever and recurrent right periorbital swelling following the recent treatment of right periorbital cellulitis and right ethmoidal sinusitis with broad-spectrum antibiotics for 2 weeks. His medical history was notable for the following: at birth, bilateral temporoparietal intraparenchymal hemorrhagic infarcts were noted with associated residual facial weakness. An extensive evaluation did not reveal any bleeding disorder or any underlying anatomic cause for his intracranial hemorrhage. He had an absolute neutrophil count (ANC) of 40 cells/µl, initially ascribed to acute illness. At 2 weeks of age, he developed a spontaneously draining abscess in his right groin. Abscess drainage culture revealed methicillin-resistant *Staphylococcus aureus* (MRSA). At that time, his ANC was 10 cells/µl. Bone marrow aspirate demonstrated myeloid maturation arrest with a few mature myeloid forms. Erythroid elements were of limited quantity and exhibited full-spectrum maturation. Megakaryocytes exhibited normal morphology. Genetic analysis was performed using targeted capture for genes associated with SCN (the University of Washington Department of Laboratory Medicine, MarrowSeq Panel, which includes ABCB7, ADA, AK2, ANKRD26, ATM, ATR, ATRX, BLM, BRCA1, BRCA2, BRIP1, C150RF41, CBL, CDAN1, CEBPA, CSF3R, CTC1, CXCR4, DKC1, ELANE, ERCC4, ETV6, FANCA, FANCB, FANCC, FANCD2, FANCE, FANCF, FANCG, FANCI, FANCL, FANCM, G6PC3, GATA1, GATA2, GFI1, HAX1, IL2RG, JAGN1, JAK2, KIF23, KLF1, LIG4, LYST, MPL, MRE11A, NBN, NHP2, NOP10, PALB2, PAX5, RAB27A, RAD50, RAD51C, RBM8A, RMRP, RNF168, RPL10, RPL11, RPL26, RPL35A, RPL5, RPS10, RPS14, RPS17, RPS19, RPS24, RPS26, RPS7, RTEL1, RUNX1, SBDS, SEC23B, SLX4, SRP72, TAZ, TCIRG1, TERC, TERT, TINF2, TP53, USB1, VPS45, WAS, and WRAP53), followed by next-generation sequencing with Illumina technology, which revealed biallelic pathogenic heterozygous mutations (p.S64X, NM_032492.3:c.191C > G and p.Q127X, NM_03492.3:c.379C > T) in the *JAGN1* gene.

**Figure 1:**
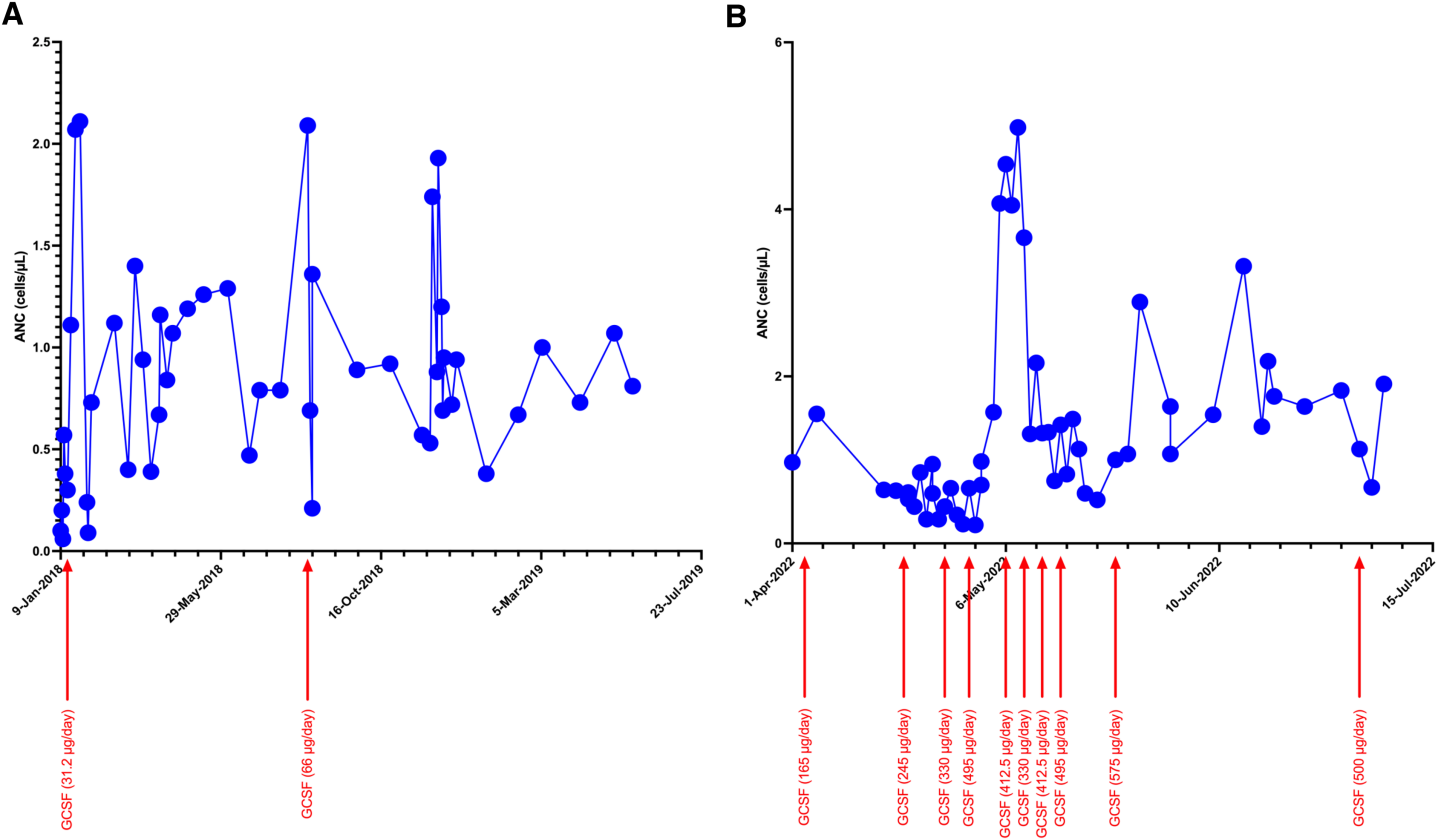
Trends of ANC during initial (**A**) and current (**B**) hospitalization with increasing doses of GCSF.

The patient was treated with granulocyte colony-stimulating factor (GCSF). However, his response to GCSF was inconsistent even with higher doses of 10 mcg/kg/day, with widely fluctuating ANC values (Figure 1). Additional screening revealed hypogammaglobulinemia with an IgG level of 53 mg/dl at 5 months of age (normal range: 200–1,200 mg/dl). Therefore, he was started on immunoglobulin replacement therapy as well. Despite these interventions, he continued to develop recurrent infections, including skin and soft tissue infections as well as chronic nasal congestion. At 1 year of age, after sustaining minor head trauma following a fall, he developed a venous hemorrhagic cerebral infarct and a retro-clival and cerebellopontine angle cistern hematoma. At 2 years of age, he experienced acute respiratory failure and bradycardic cardiac arrest attributed to an aspiration event while receiving non-invasive positive pressure ventilation after undergoing adenotonsillectomy for obstructive sleep apnea. Until the performance of the tonsillectomy, he was on continuous positive airway pressure (CPAP) for obstructive sleep apnea. In addition, he developed right parasagittal watershed infarcts between the anterior and the middle cerebral arteries with subsequent left hemiparesis. Several months later, he experienced a focal seizure and Todd's paralysis and was treated with levetiracetam for seizure prevention.

**Figure 2:**
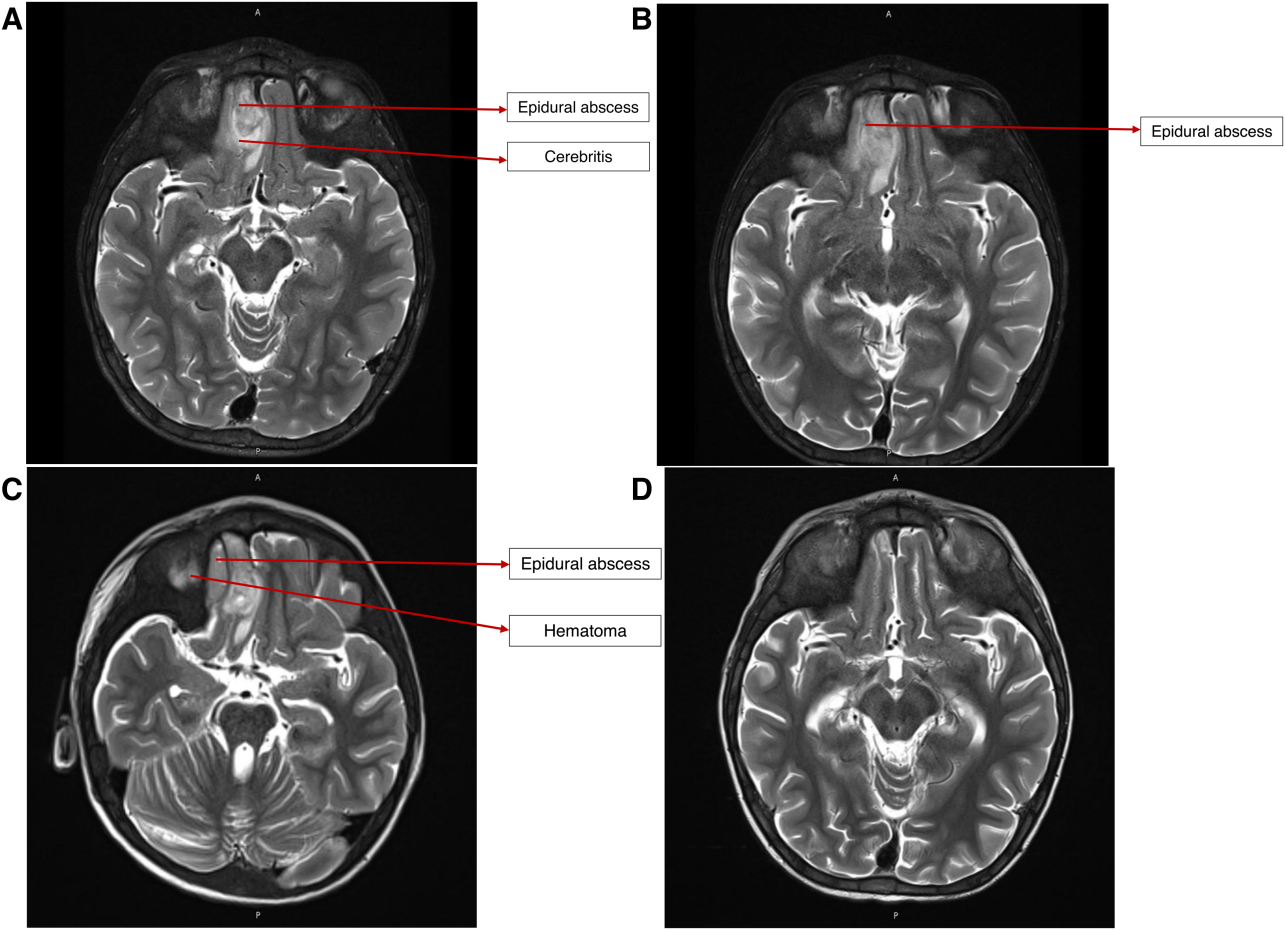
(**A**) MRI brain on presentation, demonstrating epidural abscess and reactive cerebritis. (**B**) MRI brain after endoscopic drainage procedure, showing persistence of epidural abscess and cerebritis. (**C**) MRI brain after craniotomy and debridement, showing persistence of epidural abscess and new adjacent hematoma. (**D**) MRI brain after ∼6 weeks of broad-spectrum antibiotic therapy, showing no abscess or residual parenchymal changes.

On presentation, he was alert, febrile, tachycardic, and tachypneic. He had mild right periorbital edema and erythema. Neurologic examination was significant for mild left lower facial weakness and mild left upper-extremity weakness, with slightly exaggerated reflexes on the left side globally. No dysmorphic features were observed. His height-for-age was under the 5th percentile, and his weight-for-age was between the 15th and the 25th percentile. A partially treated bacterial infection following his recent hospitalization was suspected. A brain computed tomography (CT) revealed complicated acute sinusitis with an epidural abscess. Magnetic resonance imaging (MRI) confirmed an epidural abscess with adjacent cerebritis, which was thought to be reactive in nature (Figure 2). He was hospitalized for further care.

During hospitalization, hematologic studies revealed anemia, a normal leukocyte count with neutropenia and monocytosis, thrombocytopenia, and elevated C-reactive protein (CRP) (Table 1). Initial blood cultures, cerebrospinal fluid (CSF) analysis, and CSF culture did not reveal anything significant. He was treated empirically with vancomycin, metronidazole, and cefepime. He underwent endoscopic drainage of the abscess, which demonstrated growth of *Pseudomonas aeruginosa* on culture, which was sensitive to cefepime. Nevertheless, a broad antibiotic coverage was maintained, given the inability to exclude polymicrobial infection in the context of recent antibiotic use. Achieving therapeutic vancomycin trough levels was challenging, prompting a change to daptomycin. An interval imaging demonstrated an increased size of the abscess after endoscopic surgery, and he subsequently underwent frontal craniotomy.

**Table 1:** Investigations on presentation.

Investigation	Result	Normal range
Blood profile		
Hemoglobin (g/dl)	**9.1**	11–13.7
Hematocrit (%)	**29.7**	34–44
Total WBC count (cells/µl)	7,180	4,500–13,500
Differential WBC count (%)
Neutrophil/band Lymphocyte Eosinophil Basophil Monocyte	**8.8** **44.2** **0.9** **1.8** **44.2**	55–70 20–40 1–4 0.5–1 2–8
Absolute neutrophil count (cells/µl)	**630**	1,800–8,000
Platelet count (x10^9^ cells/L)	**128**	150–450
PT (sec)	**18.1**	12.1–14.6
PTT (sec)	31.9	25.0–37.0
INR	**1.45**	0.92–1.14
Fibrinogen (mg/dl)	398	200–400
Functional antithrombin (%)	**71**	80–120
ESR (mm/h)	**23**	0–10
Urine analysis		
Glucose	Normal	
Blood	Negative	
Protein	Negative	
Ketone	Negative	
Bilirubin	Negative	
Liver function tests		
Total bilirubin (mg/dl)	0.2	0.1–1.2
Direct bilirubin (mg/dl)	<0.2	<0.3
Aspartate transaminase (U/L)	29	10–40
Alanine transaminase (U/L)	10	10–40
Alkaline phosphatase (U/L)	225	130–260
Renal function tests		
Creatinine (mg/dl)	**0.23**	0.44–0.65
BUN (mg/dl)	8	5–18
Inflammatory markers		
C-reactive protein (mg/dl)	**4.51**	<0.9
Microbiology/virology		
Blood cultures	No growth	
MRSA surveillance cultures	Negative	
Respiratory pathogen tests^a^	Negative	
Coagulation profile from 2018^b^		
Platelets (x10^3^ cells/µl)	**108**	223–461
PT (sec)	**15.4**	12.1–14.6
INR	1.19	
PTT (sec)	**37.5**	25.0–37.0
Thrombin time (sec)	15.7	14.4–18.0
Fibrinogen (mg/dl)	267	200–400
Factor II activity (%)	50	50–150
Factor V activity (%)	77	50–150
Factor VII activity (%)	66	50–150
Factor XII activity (%)	98	69–143
PTT inhibitor screen	Negative	
PT inhibitor screen	Negative	
Lupus anticoagulant screen	Negative	
vWF Risto Cofactor (%)	63	50–150
vWF Antigen (%)	83	50–160
vWF VIII (%)	79	70–170

WBC, white blood cells; PT, prothrombin time; PTT, partial thromboplastin time; INR, international normalized ratio; ESR, erythrocyte sedimentation rate; BUN, blood urea nitrogen; vWF, von Willebrand Factor.

Values outside the normal range are in bold.

^a^ Negative respiratory pathogen PCR tests for adenovirus, SARS-CoV-2, influenza A, influenza B, human metapneumovirus, parainfluenza 1, parainfluenza 2, parainfluenza 3, parainfluenza 4, respiratory syncytial virus, and rhinovirus.

^b^ After the second bleeding event.

After craniotomy, an interval MRI demonstrated a new epidural collection lateral to the epidural abscess, which was thought to represent a postoperative hematoma. A final interval MRI obtained 5 weeks after craniotomy showed a resolution of the abscess and hematoma.

Because of the nature of his surgeries, an alternative to CPAP was preferred, ultimately leading to the performance of a tracheostomy. Early during his course of stay, ANC dropped to 0, and GCSF dosing increased to ∼40 μg/kg/day to maintain an ANC >1,000 cells/µl. Because of his bleeding history, sequential compression device boots were employed while he was immobile, instead of using systemic anticoagulation.

During his hospital stay, the patient developed a febrile illness, and a blood culture taken from his central venous catheter grew *Candida lusitaniae* (*Clavispora lusitaniae*). Fungal staging examinations showed no evidence of fungal infection in CSF culture, ocular exam, or abdominal ultrasonography. His central venous catheter was removed, and he was treated with micafungin for 2 weeks.

Shortly after the patient completed a 6-week course of cefepime, daptomycin, and metronidazole for epidural abscess, his hospital course was complicated by a new requirement for supplemental oxygen and by a new retrocardiac opacity on chest x-ray, and he was diagnosed with pneumonia, which was treated with cefepime for 7 days.

His hospital stay is summarized in Table 2. On the day of discharge, his ANC was 1,910 cells/µl, and he was hemodynamically stable.

**Table 2:** Course during the hospital stay.

Day	Events
1	Presentation: pyrexia of unknown origin, moderate neutropenia. Ceftriaxone started empirically. GCSF and immune globulin therapy continued for primary immunodeficiency.
2–4	Continued fever with chronic nasal congestion and residual right eye discoloration. Partially treated periorbital cellulitis and sinusitis were suspected. Ceftriaxone was replaced with ampicillin-sulbactam. Maxillofacial CT revealed complicated acute sinusitis with frontobasal epidural abscess and adjacent cerebritis. Brain MRI confirmed epidural abscess with focal leptomeningeal enhancement. Antibiotics changed to vancomycin, metronidazole, and cefepime.
5	Source reduction—endoscopic endonasal drainage of the abscess. Pus culture: *Pseudomonas aeruginosa*. Procedural complication: CSF leak—managed by the insertion of a lumbar drain. *P. aeruginosa* sensitive to cefepime. Difficulty in achieving therapeutic vancomycin levels; vancomycin switched to daptomycin. Antibiotic regimen: daptomycin, metronidazole, and cefepime for 6 weeks.
11	Frontal craniotomy was done to manage the increasing size of the abscess. Debrided tissue culture from craniotomy: *P. aeruginosa* (sensitive to cefepime).
19	Tracheostomy was performed because of the inability to use CPAP in the context of recent maxillofacial surgeries.
21–26	New fevers. Central venous line (CVL) culture grew *Candida lusitaniae* (*Clavispora lusitaniae*)—CVL removed. LP was performed to evaluate for CNS involvement of fungal infection: no fungal elements or pleocytosis noted. Ophthalmologic exam reassuring. Antifungal added: micafungin for 2 weeks.
45–46	Transferred out of ICU in stable condition. Remains with tracheostomy in place. Interval brain MRI shows a resolution of intracranial infections. Broad-spectrum antibiotic course terminated.
58–70	Recrudescence of fever with worsening supplemental oxygen requirement and worsening abdominal distention. Chest x-ray: new retrocardiac opacity. Abdominal ultrasound showed acute appendicitis. Tracheostomy tube aspirate culture: *P. aeruginosa*. Received 7 days of cefepime for pneumonia, and was transitioned to ceftriaxone and metronidazole for a 14-day course because of the finding of appendicitis. Resolution of fever, with the persistence of tachycardia. Cardiology consultation: likely benign tachycardia. *Pneumocystis jirovecii* prophylaxis with trimethoprim-sulfamethoxazole initiated.
**82**	Discharged home.

He was readmitted approximately 2 months after discharge for planned sibling donor allogeneic hematopoietic stem cell transplantation.

## Discussion

Herein, we describe a rare case of a patient with SCN associated with biallelic *JAGN1* mutations. SCN is uncommon, with associated genetic mutations occurring in >20 genes, including *ELANE, GFI1, HAX1, G6PC3, WAS,* and *VPS45*, and diverse inheritance patterns that can be autosomal-dominant, autosomal-recessive, or X-linked (5, 6). SCN caused by JAGN1 deficiency is extremely rare, accounting for ∼10% of cases (5).

**Figure 3:**
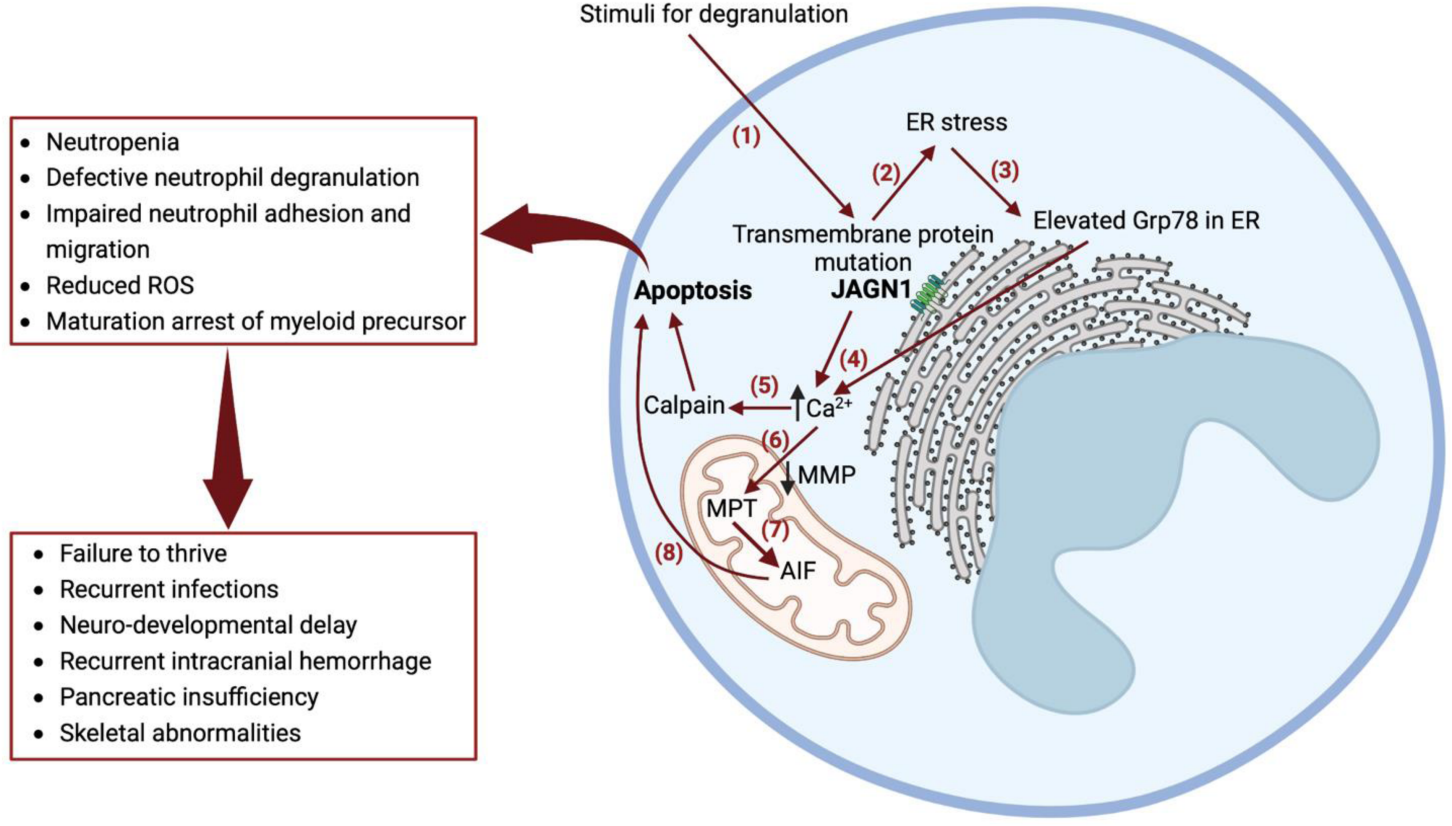
Mechanism of severe congenital neutropenia caused by JAGN1 deficiency. (1) Stimuli for degranulation in a wild-type JAGN1 transmembrane protein produces degranulation. (2) Mutant JAGN1 produces ER stress. (3) ER stress results in increased Grp78 protein. (4) Elevated Grp78 causes N-glycosylation. (5) Altered N-glycosylation results in elevated calcium in the cytoplasm, causing activation of calpain, which leads to apoptosis. (6) Reduced MMP leads to Ca^2+^ entry to mitochondria, causing MPT. (7) MPT causes stimulation of AIF, which is cleaved by calpain. (8) AIF released in cytoplasm activates programmed cell death (apoptosis). Grp78—immunoglobulin heavy-chain binding protein of heat shock protein 70 family, a regulator of unfolded protein response, which is induced in cells with endoplasmic reticulum stress. They act as a molecular chaperone and a key regulator of Ca^2+^ homeostasis; GM-CSF improves N-glycosylation and is therefore used as a treatment for congenital neutropenia in JAGN1 mutation. MMP, mitochondrial membrane potential; AIF, apoptosis-inducing factor; JAGN1, jagunal homolog 1 membrane protein; MPT, mitochondrial permeability transition; Ca^2+^, calcium ion; ROS, reactive oxygen species.

JAGN1 is expressed in the endoplasmic reticulum (ER), which contributes to the early secretory pathway in the ER and is a critical regulator of neutrophil differentiation and survival via GCSF receptor-mediated signaling (4). Biallelic mutations in *JAGN1* lead to several defects in the granulocyte structure and cellular function and also affect longevity (4). A knockdown of JAGN1 expression in HeLa cells interferes with STAT3 phosphorylation upon recombinant human GCSF treatment, suggesting that decreased GCSF receptor signaling may contribute to defective granulocytes (4). Granulocyte-macrophage colony–stimulating factor (GM-CSF) treatment of bone marrow granulocytes in patients with JAGN1 deficiency restores the phosphorylation of STAT5 and cytotoxicity in response to *Candida albicans* (7). JAGN1 deficiency is also associated with a decreased expression of myeloperoxidase (MPO) in neutrophils, contributing to an ineffective killing of *C. albicans* via neutrophil extracellular traps—a phenotype reversible via GM-CSF administration (8). Mutations in *JAGN1* can result in ER stress and intracellular calcium activation of calpain, leading to myeloid cell apoptosis (Figure 3) (9).

Similar to the case of our patient, who had significantly low IgG levels by 5 months of age, at least two other patients with JAGN1 deficiency have been described as having antibody deficiency (10). In a murine model, JAGN1-deficient B cells had defective antibody production and secretion, altered immunoglobulin glycosylation, and defects in the differentiation and maintenance of plasma cells (11). These findings have been attributed to increased ER stress and dysregulation of the unfolded protein response. Notably, even JAGN1-deficient patients with normal serum immunoglobulin levels demonstrate an altered immunoglobulin glycoprofile when compared with healthy donors, suggesting the need for a very low threshold for immunoglobulin replacement in all patients with JAGN1 deficiency, regardless of the IgG levels (11).

SCN caused by JAGN1 deficiency may be associated with recurrent intracranial hemorrhage, pancreatic insufficiency, failure to thrive, developmental delay, skeletal abnormalities, and recurrent infections (including fungal infections) in the context of erratic neutrophil counts despite treatment with GCSF. In such cases, hematopoietic stem cell transplantation may be considered and is curative (4).

A summary of published case reports with SCN caused by JAGN1 deficiency is presented in Table 3. The case of an index family in Northern Africa was reported, but few details were provided (5). Of the other reported cases, the majority were females either from Turkey or Algeria. The mean age was 10 years and the mean ANC at the time of presentation was 100 cells/µl. Common clinical features associated with the mutation were sepsis, abscess, pneumonia, failure to thrive, and neurodevelopmental delay, and genetic defects were homozygous mutations in exons 1 and 2 of *JAGN1* in addition to the alteration of proteins.

**Table 3:** Case reports of severe congenital neutropenia caused by the JAGN1 mutation.

	Patient	Sex	Country	Clinical features	Investigations	Genetic defect	Treatment and Outcome
1.	10-year-old (Y) (12)	M	Turkey	Facial dysmorphism; neutropenia; cough; rhinorrhea; neonatal sepsis; recurrent skin ulcers and abscesses; recurrent pneumonia, otitis media, sinusitis; cavernous lesions; asthma; allergic conjunctivitis	ANC: 100/mm^3^; leukocyte count: 5,120/mm^3^; Hb: 10.9 g/dl; platelet count: 357,000/mm^3^; bone marrow: maturation arrest of neutrophils	Homozygous mutation c.130 c > T in JAGN1 gene; p.His44Tyr protein alteration	GCSF (5 µg/kg)
2.	5 children from index family (5)	—	Northern Africa	Failure to thrive, developmental delay, skeletal abnormalities	—	Homozygous mutation c.3G > A in JAGN1 gene	—
3.	23-Y (4)	F	Algeria	ENT infections, aphthosis, perianal cellulitis, skin abscesses	ANC: 830/µl; bone marrow: maturation arrest	Homozygous mutation c.3G > A in JAGN1 gene; p.Met1lle protein alteration	Poor response to GCSF; alive
4.	17-Y (4)	F	Algeria	ENT infections, short stature	ANC: 800/µl	Homozygous mutation c.3G > A in JAGN1 gene; p.Met1lle protein alteration	Poor response to GCSF; alive
5.	19-Y (4)	M	Algeria	Aphthosis, skin abscesses, balanitis, pneumonitis, lung abscess, osteitis, perianal cellulitis, pyloric stenosis	ANC: 570/µl; bone marrow: maturation arrest (intermittent)	Homozygous mutation c.3G > A in JAGN1 gene; p.Met1lle protein alteration	Poor response to GCSF; alive
6.	17-Y (4)	F	Algeria	Otitis, paraodontopathy, scoliosis, dental malformations	ANC: 501/µl; bone marrow: maturation arrest (intermittent)	Homozygous mutation c.3G > A in JAGN1 gene; p.Met1lle protein alteration	Poor response to GCSF; alive
7.	5-Y (4)	M	Algeria	ENT infections, aphthosis, skin abscesses, pneumonitis, lung abscess, perianal cellulitis	ANC: 165/µl; bone marrow: maturation arrest	Homozygous mutation c.3G > A in JAGN1 gene; p.Met1lle protein alteration	Poor response to GCSF; alive
8.	12-Y (4)	F	Iran	Upper respiratory tract infections, pneumonia, skin abscesses, febrile convulsion, focal epilepsy	ANC: 892/µl; bone marrow: maturation arrest	Homozygous mutation c.59G > A in JAGN1 gene; p.Arg20Glu protein alteration	Poor response to GCSF; alive
9.	10-Y (4)	M	Turkey	Upper respiratory tract infections, pneumonia, skin and perianal abscesses, sepsis (*Haemophilus influenzae*), extramedullary hematopoiesis with a thickening of the skull bones	ANC: 191/µl; bone marrow: maturation arrest	Homozygous mutation c.130C > T in JAGN1 gene; p.His44Tyr protein alteration	Poor response to GCSF; alive
10.	7-Y (4)	F	Turkey	Upper respiratory tract infections, skin abscesses, B/L hip dysplasia, extramedullary hematopoiesis with a thickening of the skull bones	ANC: 3,587/µl; bone marrow: maturation arrest	Homozygous mutation c.130C > T in JAGN1 gene; p.His44Tyr protein alteration	Poor response to GCSF; alive
11.	28-Y (4)	F	Iran	Skin abscesses, onycholysis	ANC: 920/µl; bone marrow: maturation arrest	Homozygous mutation c.40G > A in JAGN1 gene; p.Gly14Ser protein alteration	Poor response to GCSF; alive
12.	13-Y (4)	M	Israel	Aspergillosis, severe osteoporosis, repeated bone fractures	ANC: 130/µl; bone marrow: maturation arrest	Homozygous mutation c.297C > G in JAGN1 gene; p.Tyr99* protein alteration	HSCT; alive
13.	5-Y (4)	F	Morocco	Skin abscesses, omphalitis, pancolitis, lipomatosis, pancreatic insufficiency, bone abnormalities, dental malformations	ANC: 70/µl; bone marrow: maturation arrest (intermittent)	Homozygous mutation c.485A > G in JAGN1 gene; p.Gln162Arg protein alteration	Died (pancolitis and septicemia)
14.	16-Y (4)	F	Albania	Skin abscess, upper respiratory tract infections, pneumonia, short stature, amelogenesis imperfecta, neurodevelopmental delay	ANC: 408/µl; bone marrow: maturation arrest	Homozygous mutation c.63G > T in JAGN1 gene; p.Glu21Asp protein alteration	Poor response to GCSF; alive
15.	<1-month-old (4)	F	Pakistan	ENT infections, upper respiratory tract infections, pneumonia, sepsis (*Escherichia coli*), failure to thrive, coarctation of the aorta, mild developmental delay	ANC: 290/µl; bone marrow: maturation arrest, slight dyserythropoiesis	Homozygous mutation c.485A > G in JAGN1 gene; p.Gln162Arg protein alteration	HSCT; alive
16.	25-Y (4)	F	Germany	Pneumonia, bronchiectasis	ANC: 128/µl; bone marrow: maturation arrest	Homozygous mutation c.35_43delCCGACGGCA in JAGN1 gene; p.Thr12_Gly14del protein alteration	HSCT; alive
17.	9-months-old (10)	M	Turkey	Gluteal abscess, hepatomegaly, cervical lymphadenopathies, dysmorphic face, failure to thrive, developmental delay, hypospadias, left undescended testis, pneumonia, diarrhea, otitis, gingivitis, B/L bronchiectasis	ANC: 136/mm^3^; leukocyte count: 10,528/mm^3^; bone marrow: maturation arrest at the promyelocyte/myelocyte stage, reduced mature neutrophils	Homozygous mutation c.130C > T in JAGN1 gene; p.His44Tyr protein alteration	IVIG (0.5 g/kg), GCSF (5 µg/kg); alive
18.	4 months old (10)	F	Turkey	Dysmorphic face, amelogenesis imperfecta, gingival hypertrophy, short stature, recurrent skin abscesses, otitis, pneumonia, mild learning disability	ANC: 300/mm^3^; leukocyte count: 2,000/mm^3^; monocytes: 1,500; bone marrow: maturation arrest at the promyelocyte/myelocyte stage with mild nuclear dysplasia	Homozygous mutation c.130C > T in JAGN1 gene; p.His44Tyr protein alteration	IVIG, GCSF; alive
19.	2-Y (13)	M	Romania	Dysmorphic face, convergent monocular strabismus (syndrome of Stilling–Turk–Duane type 1), moderate growth, 2/6 systolic murmur, multiple B/L inguinal abscesses, recurrent respiratory infections, pneumonia, otitis, oral and genital candidiasis, recurrent skin abscesses	ANC: 2,400/mm^3^; CRP: 1.04 mg/dl; wound swab positive for multiresistant *Pseudomonas aeruginosa* and *Klebsiella pneumoniae*; bone marrow: absence of mature myeloid cells	Homozygous mutation c.G63T in JAGN1 gene; p.Glu21Asp protein alteration	GCSF; intravenous broad-spectrum antibiotics; oral and topical antifungals; surgical curettage
20.	2-Y (9)	M	Iraq	Umbilical infection, recurrent bronchitis, pneumonia, neutropenia, severe periodontitis, secondary diabetes mellitus, liver GVHD	ANC: < 0.5 × 10^9^/L; bone marrow: maturation arrest of myelopoiesis; pro-LL-37 absent in plasma	Homozygous mutation c.40G > A in JAGN1 gene; p.Gly14Ser protein alteration	Recombinant GCSF
21.	6-Y (9)	M	Roma origin	Neutropenia, recurrent bacterial infections, nephrolithiasis	ANC: < 0.5 × 10^9^/L; bone marrow: maturation defect (granulocytic series left-shifted to metamyelocytes); antigranulocytic antibodies absent	Homozygous mutation in JAGN1 gene; p.Glu21Asp protein alteration	GCSF
22.	2-Y (9)	M	Roma origin	Neutropenia, monocytosis, recurrent and severe bacterial infections, chronic gingivitis, mouth ulcers, short stature, failure to thrive, severe periodontitis, loss of teeth	ANC: 0.06 to 0.6 × 10^9^/L; bone marrow: maturation arrest at the myelocyte stage	Homozygous mutation in JAGN1 gene; p.Glu21Asp protein alteration	GCSF (poor tolerance), antibiotic prophylaxis

M, male; F, female; Hb, hemoglobin; JAGN1, jagunal homolog 1; ENT, ear nose throat; B/L, bilateral; HSCT, hematopoietic stem cell transplant; IVIG, intravenous immunoglobulin; GVHD, graft vs. host disease.

Our patient shared some common clinical features with cases reported in the literature, such as recurrent infections during hospital stay. To the best of our knowledge, this is the first report of recurrent intracranial hemorrhage occurring after minimal trauma among patients with *JAGN1* deficiency. Our patient was subjected to a thorough evaluation for discovering possible etiologies for his bleeding diathesis. This involved an extensive evaluation for any underlying coagulopathy that was negative (Table 1) as well as platelet function assays. In addition, imaging studies did not reveal any arteriovenous malformations. Despite this thorough outpatient investigation, the etiology of bleeding diathesis remains unknown. It is possible that *JAGN1*, which enhances glycosylation of neutrophil proteins, has as-yet unknown effects on platelet or coagulation factor function as well.

## Conclusion

In this study Here, we report that an invasive epidural abscess caused by *P. aeruginosa* occurred in a patient with SCN secondary to *JAGN1* mutation, who initially presented with periorbital cellulitis. Given the variability seen in the clinical presentation of patients with SCN secondary to JAGN1 deficiency, a high index of suspicion for invasive pyogenic infection should be maintained in those with this immunodeficiency. This should lead to prompt initiation of treatment and prophylaxis for life-threatening infections, especially when patients with SCN secondary to *JAGN1* mutation present with new focal neurologic defects, altered mental status, or have new abnormalities noted during physical examination. We also report a history of recurrent intracranial hemorrhage in our patient, which is considered unusual. Bleeding diatheses can be considered a contributor to any new symptom or finding in patients with this condition, and clinicians should consider providing anticipatory guidance and precautions to such patients, which will help achieve the goal of trauma prevention.

## Data Availability

All relevant clinical data are included in the article. Further inquires can be directed to the corresponding author.
